# Ovarian Microbiota, Ovarian Cancer and the Underestimated Role of HPV

**DOI:** 10.3390/ijms232416019

**Published:** 2022-12-16

**Authors:** Massimiliano Cazzaniga, Marco Cardinali, Francesco Di Pierro, Alexander Bertuccioli

**Affiliations:** 1 Scientific & Research Department, Velleja Research, 20125 Milano, Italy; 2 Department of Internal Medicine, Infermi Hospital, AUSL Romagna, 47921 Rimini, Italy; 3 Digestive Endoscopy Unit and Gastroenterology, Fondazione Poliambulanza, 25124 Brescia, Italy; 4 Department of Biomolecular Sciences, University of Urbino Carlo Bo, 61122 Urbino, Italy

**Keywords:** cancer, microbiota, HPV, lactobacillus, lentinula edodens, AHCC

## Abstract

In recent years, many studies have highlighted the possible close correlation between human diseases and definite patterns of microbial organisms colonizing various organs. Even at sites traditionally considered sterile, such as the upper female reproductive tract (FRT), it is now well-recognized as hosting a low biomass of different bacterial phyla. Additionally, the data from recent studies highlight a possible link between lower and upper FRT dysbiosis with a potential predisposition to cervical and ovarian cancer. Acinetobacter, chlamydia, increased mycoplasma, and lactobacillary scarcity in the upper FRT have all been linked to a predisposition to ovarian cancer. Additionally, a high-diversity vaginal community state type (CST) is linked to the presence and persistence of high-risk human papillomavirus (HPV), resulting in decreased cellular p53 activity and a reduction in the immune activity of T lymphocytes, resulting in cervical and ovarian cancer predisposition. While these findings are still far from being clarified in all aspects, in patients with multiple risk factors for ovarian cancer, a *Lactobacillus crispatus* treatment with a product with a proven ability to restore a favorable CST should be considered as an add-on therapy.

## 1. Introduction

**Figure 1:**
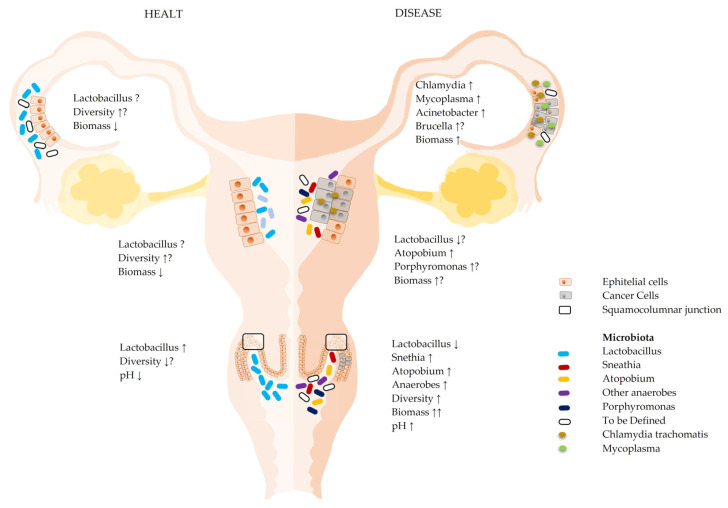
Microbial communities at various levels of the reproductive system and their variation with healthy and diseased states. Microbial composition and other characteristics of the microenvironment in healthy and pathological states in the various areas of the female reproductive system (FRT). Healthy vaginal microbiotas are mainly composed of low microbial biodiversity and are dominated by lactobacilli. In contrast, the biomass of the upper reproductive system (endometrium, tubal, and ovary) may have a much lower biomass and a higher biodiversity than the cervical microbiota, with a lactobacillary share that decreases further inside the reproductive system. In pathological conditions, the composition and biomass of the microbiota from the entire reproductive system change considerably (see text) [4]. The arrows represent increase or decrease depending on the orientation, the question marks indicate that the issues are still to be definitively clarified.

The microbial influence on different pathological scenarios is caused by the complex relationship between two different situations that are closely interconnected. The first situation concerns the intestinal microbiota and the corresponding genomic contribution, which directly or indirectly affects numerous physiological and pathophysiological functions of the organism due to its important biodiversity (both quantitative and qualitative) [1]. The second situation involves the influence of the “local” microbiota, the set of microbial populations that populate the various organs and systems, on the same functions described above [2]. In gynecological pathologies, the focus is usually on the involvement and action of the vaginal microbiota. However, in light of the recent progress, it is now evident that, regarding the non-sterility of some gynecological areas, it is more accurate, complete, and scientifically necessary to speak of “the microbiota of the female reproductive tract (FRT)” [3]. They can be broadly divided into the microbiota of the lower and upper reproductive systems (the lower and upper FRT) [4]. Regarding the “higher” gynecological areas, there is a constant decrease in the lactobacillary share and the simultaneous occupation of these biological niches by different bacterial phyla, such as the proteobacteria species Actinobacteria and Bacteroidetes, which, under conditions of pathology, are particularly represented by the increase in potentially pathogenic species, most commonly Sneathia, Atopobium, Porphyromonas, *Chlamydia trachomatis*, Brucella, and Mycoplasma (Figure 1) [5]. All abbreviations are reported in Table 1.

### 1.1. Microbiota of the Lower Female Reproductive Tract (Lower FRT)

The microbiota of the lower female reproductive system is substantially represented by the vaginal microbiota, which can be classified according to “community state type”, “vaginotypes”, or, more appropriately, “stable microbial consortia” (CST) [6]. The vaginal microbial communities of healthy women of childbearing age are grouped into five different categories, four of which are characterized by the presence of Lactobacilli, and each of which can be traced back to a single species with a very low biodiversity. Particular attention is given to CST I dominated by *Lactobacillus crispatus*, considered the most stable and clinically important microbial consortium with the greatest potential for improving health. In addition to the commitment of the biological niche, the protective role of Lactobacilli is associated with the production of lactic acid (in particular protonated with virucidal and bactericidal capacity), thus maintaining a relatively low pH and Nugent score [7]. In contrast, CST IV is considered the consortium most correlated with a pathological state. It has a very low lactobacillary content and a greater biodiversity that includes bacterial species, in particular anaerobic species, some of which are potentially pathogenic [8]. CST IV is frequently associated with bacterial vaginosis, a condition prodromal to the onset of numerous gynecological diseases, such as sexually transmitted diseases, preterm birth, infertility, spontaneous abortion, pelvic inflammatory diseases, endometriosis, and some cancers [8,9].

### 1.2. Microbiota of the Upper Female Reproductive Tract (Upper FRT)

The microbiota of the upper reproductive system is very different in terms of both biomass and biodiversity, which appears to be the consequence of a modification/evolution of the microbial population as we move toward the deeper organs of the reproductive system. Recent data [4] show that the biomass of the upper reproductive system’s microbiota is about 10,000 times lower than that of the vaginal area. However, it has considerably greater biodiversity since the share of Lactobacilli exponentially decreases in deeper parts of the reproductive system (leaving room for other species with more varied bacterial populations: 97% at the cervical level; 30% at the uterine level; and 1.7% at the tubal and ovary level). It is important to underline that these data are likely the result of some bias since they were obtained from tissue biopsies (in particular from women with infertility problems) and could therefore be the tissue of potentially unhealthy subjects or those with health conditions. Despite this and a range of different results, the literature is now in agreement that there is a trend of microbial evolution in the female reproductive system. Therefore, this trend must always be considered in pathological and preventive terms.

## 2. Microbiota and Ovarian Cancer

According to a classification system that is now universally accepted from the histological point of view, ovarian tumors are divided into epithelial (about 65%), stromal (about 10%), and germinal (about 20%), with a peak incidence between 50 and 60 years of age, in peri- or postmenopausal age [10]. Factors such as reproductive history, endocrine factors, overweight and obesity, and smoking habits play important roles in cancer incidence [11]. In recent decades, genetic analysis techniques have shown that the presence of genetic mutations in some tumor suppressor genes, such as BRCA 1 and 2, as well as p53, are responsible for a large proportion of ovarian oncological pathologies, in particular those that occur at an early age compared to the typical age of onset [12].

Regarding gynecological cancer pathogenesis, other factors were recently investigated. The findings on gut microbiota show that the presence of some species is considered part of the development of non-gynecologic oncological pathologies through chronic inflammation, such as *Helicobacter pylori* related to gastric adenocarcinoma, *Streptococcus bovis* related to colorectal adenocarcinoma, and *Salmonella enterica* serovar typhi related to cholangiocarcinoma [13]. Therefore, in gynecological cancers, some bacterial species could play important roles in pathogenesis.

Initial evidence highlights how alterations within the microbiota of the gastrointestinal tract and female reproductive tract may promote a pro-carcinogenic condition by modifying the host’s immune response and hormone metabolism and intervening in cell cycle processes and apoptosis. Certain microbes have demonstrated DNA damage capability, and they could directly induce cell apoptosis with the processing of pro-apoptotic substances or indirectly with the production of reactive oxygen species [13]. In cervical cancer pathogenesis, cervical and vaginal microbiota have increasingly been considered as mediators of the persistence and carcinogenesis of human papillomavirus (HPV) infection. Specifically, the vaginal microbiota of women who had not regressed from HPV infection during a follow-up study correlated with higher proportions of anaerobic bacteria, such as *Prevotella timonensis* and *Gardnerella vaginalis*, compared to those who had regressed and showed higher levels of Lactobacillus species [13]. A small number of studies considered the probiotic administration of Lactobacillus strains in HPV-infection-affected women as a strategy to prevent HPV persistence and cervical cancer carcinogenesis [14,15]. Although preliminary studies are encouraging, further research is still required.

Recent scientific acquisitions in the microbiological field highlighted the importance of microbiota, and vaginal and ovarian cancers as real risk factors, considering dysbiosis understood as a numerical and/or qualitative alteration of a microbial population and as a non-secondary element in the history of ovarian cancer [13].

## 3. Vaginal Microbiota and Ovarian Tumor

**Figure 2:**
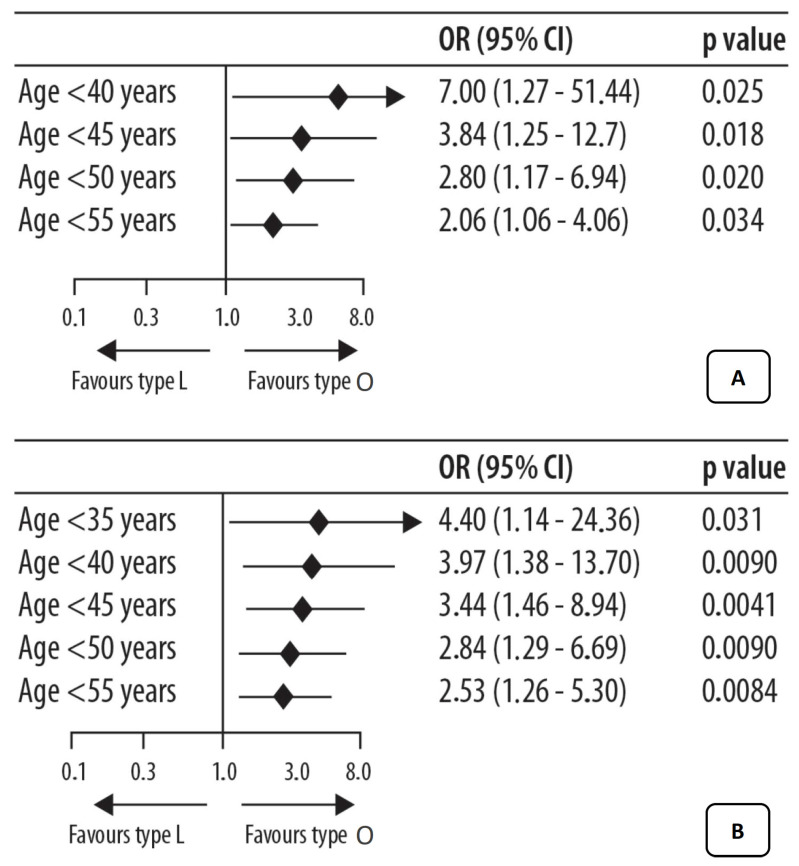
Prognostication of CST for age cluster in ovarian oncological pathology set: (**A**) with ovarian oncological pathology status as the predictor, (**B**) in the BRCA set (mutation status) as the predictor [16]. As discussed in the text, these data suggested a greater influence in relation to the age of the patients, with a progressively greater relationship in younger ages.

By analyzing the influence of the vaginal microbiota in women with ovarian cancer or at a high risk of developing this cancer (BRCA1+ mutation or high probability of mutation), an altered and characteristic vaginal microbiome can be found [16]. Patients were divided into affected and mutated healthy patients and underwent an analysis of the vaginal microbiota to identify microbiota that had a share of Lactobacilli >50% (patients with CTS I, II III, and V identified globally as group “L”) and Lactobacillary <50% (CTS IV patients with a predominance of anaerobic bacteria, in particular, Gardnerella identified as the group “O”). It emerged that the presence of ovarian cancer or known predisposing factors (BRCA1 mutation, age, etc.) is seemingly associated with an altered and unfavorable local bacterial community, as it is low in Lactobacilli (community O), suggesting a close relationship between a specific CST (or a specific microbial population) and the possibility of developing the disease. Furthermore, lactobacillary scarcity was found to be stronger and more marked in younger patients (<50 years) at risk of or suffering from an overt disease (Figure 2).

Caution must be taken when considering these factors. Despite the innovative and authoritative research of Nenè et al., at present it is not possible to clinically define whether the use of aids to facilitate bacterial colonization really manages to restore favorable microbiota. Additionally, if it were to succeed, it is unclear whether the restoration of a favorable CST would actually lead to a reduction in the risk of ovarian cancer [16]. Future studies will clarify the effective clinical role in these aspects.

## 4. The Ovarian Microbiota and Ovarian Cancer

The presence of an ovarian microbiome, its exact composition, its dysbiosis that could accompany the onset and progression of some adnexal diseases, and the involved biological mechanisms are very new topics and remain far from being clarified in all aspects.

### 4.1. The Role of Bacteria

**Figure 3:**
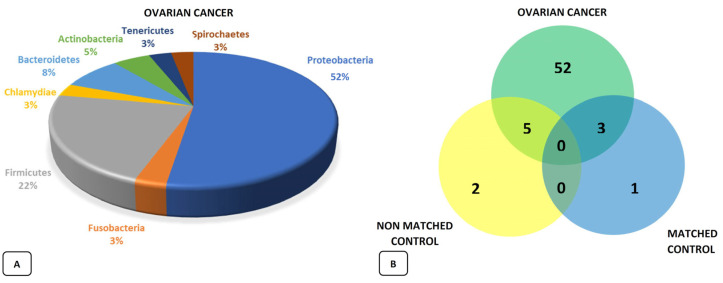
Bacterial signature identified in ovarian K-tissue: microbial composition in tumor tissues (**A**); Venn diagram with the evident preponderance of bacterial species only present in pathological tissues (**B**) [22].

Recent studies have highlighted and confirmed the presence of a specific ovarian microbial population, and researchers are driving themselves to identify a real “signature” that is apparently associated with the onset of cancer [17]. In fact, it is possible that the microbial population and tumor influence each other under the assumption that a specific tumor microenvironment may be ideal for a particular microbial population, ensuring an optimal niche for its growth and biological influence or that a certain microbial scenario is, through its peculiar biological functions, the ideal substrate for the growth and development of cancer cells [18]. This allows us to hypothesize about the presence of specific microbiomes associated with various types of cancer, as well as the presence of related biomarkers and targets, which can be used for their diagnosis, prognosis, and prevention. The analysis of ovarian tumor tissues highlighted the modification of the Proteobacteria/Firmicutes ratio, with an increase in the former and a significant increase in the Acinetobacter species. The study by Zhou et al. [19] presented interesting data when compared with the findings of Shanmughapria et al. [20]—a chlamydia infection (with consequent inhibition of apoptosis and DNA damage) in 70% of the analyzed tumor tissues—and by Chan’s working group, who reported the presence of mycoplasma in 59% of cases [21]. A more recent analysis of the microbial population conducted in 99 ovarian tumor samples (bacteria, viruses, fungi, and parasites) found that—compared to observations in 20 samples from healthy tissues adjacent to the tumor (defined matched) and in 20 other “non-matched samples” from the healthy tissue of unaffected patients—the existence of a real “ovarian microbiota” with an increase in biodiversity but a difference in microbial population between healthy and disease-affected tissues. This led to the identification of the exclusive presence of 52 bacterial agents only in the tumor tissue, with a particular predominance of the Proteobacteria phylum present in more than half of the analyzed samples (Figure 3) [22]. What these various papers seem to suggest is the presence of an amplified and highly variable bacterial share (greater biodiversity) in all tumor tissues compared to those used as a control, demonstrating the direct and important influence of the microbial population on the development and progression of a local-oncological pathology.

### 4.2. The Role of Viruses

**Figure 4:**
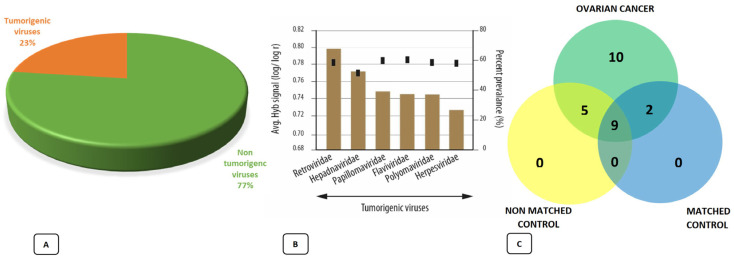
Viral signature found in ovarian tumor tissues: 23% of the discovered viruses were of the tumorigenic type and were found in more than 50% of the tumor samples (**A**). Major viral groups represented (**B**). The Venn diagram highlights how most of the viral findings are exclusively relevant to pathological tissues (**C**) [22].

This situation has also been highlighted in differential viral expression between tumor and healthy tissues, suggesting the possibility that viruses are also implicated in the carcinogenic process and tumor progression [22]. The analysis found that among all the discovered viral families, as much as 23% were represented by potentially tumorigenic viruses and that these were present in more than 50% of the analyzed tumor tissues. The largest share was that of Retroviridae, with the significant presence of some herpesviruses and human papillomavirus. HPVs were found in tumor tissue but not in adjacent healthy tissue and were mostly represented by high-risk HPV 16 and 18. This seems to be generally confirmed by the hypothesis of a relationship between viral presence and oncological pathology, emphasizing the role of a couple of particularly involved viral elements, such as a human herpesvirus-6a (HHV-6a) and HPV (Figure 4).

## 5. The Possible Role of HHV-6a in Ovarian Cancer

HHV-6a is present in more than 50% of tumor samples and manifests its oncological potential with two characteristic mechanisms. The first mechanism is its ability to block the activity of insulin-like growth factor-binding proteins (IGFBPs), resulting in a greater share of this free and active growth factor with potentially mitogenic consequences [23]. The second mechanism seems to activate some oncogenic genes, such as SH3RF2 (which normally controls the activity of the p21 protein), thus facilitating the clinical expression of some mutations that would normally remain silent [23].

## 6. The Possible Role of HPV in Ovarian Cancer

Several pieces of data appear to confirm the presence of a close relationship between viral infection, in particular for some high-risk HPV serotypes (16 and 18 at first), and the presence of pathology, also signaling the possibility of the presence of other types of viral infections such as cytomegalovirus (CMV), often associated with HPV. This underlines the relationship between infection and the stage of the disease, with a share of viral presence that increases as the disease becomes more advanced, elucidating its role not only in terms of onset but also prognosis [23,24,25]. In confirming this hypothesis, some studies found a significant reduction in the p53 protein in HPV+ tumor tissues. Given the ability of the oncoproteins produced by the virus (E6 and E7) to bind and inhibit the activity of some tumor suppressor genes, including p53, this is not only plausible but potentially decisive in carcinogenetic and prognostic mechanisms, given that the inhibition of p53 also seems to be more present in tumors with a worse prognosis [26,27]. The presence of HPV, even with a high risk in the cervical area, is a potential but insufficient condition for the development of the disease since a spontaneous regression of the infection is observed [28]. Conditions that favor the persistence of HPV by increasing its oncogenic power, such as the immunosuppression of T lymphocytes, are vital. The reduced immune activity of the host makes it difficult to fight the infection, favoring its persistence and, thus, the expression of its pathological potential [29]. This fundamental pathophysiological condition at the cervical level could also prove to be relevant at the ovarian level, as suggested by a retrospective study where just under 200 tissues of high-grade ovarian tumors (III and IV) were analyzed. Following the evaluation of the lymphocyte infiltration rate (able to highlight an inadequate immune response) in relation to the outcome of the disease, it emerged that significantly lower mortality occurs in subjects with lymphocyte presence. Therefore, immune activity against the tumor was even greater in patients who fully responded to chemotherapy, highlighting a role in therapeutic response [30]. The effect of the presence and progress of adequate immune activity toward disease conditions was highlighted. The way that the disease likely responds to the same pathogenetic mechanisms at the cervical and ovarian level, with an evident and fundamental dependence on an immune response also conditioned by a specific viral population, was also analyzed.

**Figure 5:**
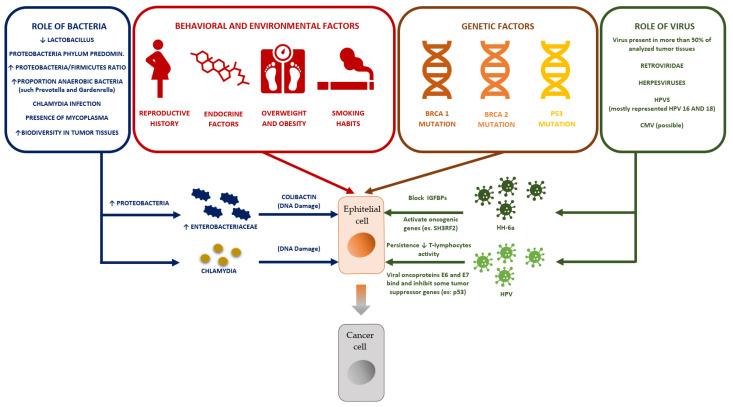
Summary of mechanisms potentially involved in the development of ovarian cancer: environmental and behavioral factors; genetic factors; the role of bacteria; the role of viruses (see text).

All the mechanisms described in the previous paragraphs are summarized in Figure 5.

## 7. Possible Therapeutic Scenarios

From the discussions on this topic (relevant research findings reported in Table 2), it is clear that, together with the known risk factors for ovarian cancer (reproductive history, endocrine factors, overweight and obesity, and smoking habit), it is also necessary to consider the influence of vaginal and ovarian microbiota (and their relationships) and fluctuating physiological and pathological activities depending on the disease type and condition of a patient.

The known risk factors must be addressed with appropriate interventions that aim to address sedentary lifestyles, nutritional dynamics, and unhealthy habits in patients [31,32]. The unfavorable microbial conditions affecting tumor onset and progression that can be managed and possibly corrected in some cases can be summarized as follows:

(1) Presence of an unfavorable vaginal CST (CST III or IV);
(2) Presence and persistence of high-risk HPV with decreased p53 activity;
(3) Reduction in immune activity expressed by the reduced proportion of T lymphocytes.

The attempt to restore an adequate CST for ovarian pathology can be carried out by administering Lactobacilli, favoring not only the displacement from an unfavorable CST but also the colonization by Lactobacilli, which is particularly favorable for the production capacity and quality of the produced lactic acid and its anti-inflammatory properties. From this perspective, the Crispatus species of Lactobacilli (which identifies CST I) can be considered therapeutically preferable. As described by DiPierro et al. for the *Lactobacillus crispatus* strain M247 (LMGP-23257), a 3-month treatment with at least 20 billion colony-forming units (CFU), modified almost all CSTs towards a type I CST, which is favorable in patients with persistent HPV infection (sexually active), contributing to the eradication of about two thirds of present high-grade HPV [33]. The product used was made by Labomar SpA (Istrana, Treviso, Italy), marketed by Pharmextracta SpA (Pontenure, Piacenza, Italy), and notified to the Authority Sanitaria Italiana on 1 March 2019 with the notification number 115,450 (commercial name: Crispact ^®^).

With these data in mind, it is important to note that, for many *Lactobacillus crispatus* based products marketed in Italy, there is a discrepancy between the declared dose and the dose that is actually present [34], casting doubt on the ability of these products to modify the vaginal microbiota. HPV eradication could also be attempted with the administration of alpha-glucans, short-chain and low-molecular-weight polysaccharides (even simultaneously). Several substances of plant origin have been investigated over time for common clinical [35] and oncological applications [36]. The literature shows how a particular mixture of alpha-glucans called Active Xerose Correlated Compound (AHCC), obtained from the fermentation of a primary mycelium of a fungus (*Lentinula edodes*), is able to act both by promoting the elimination of the virus (increasing the levels of interferon γ known to be anti-viral and simultaneously decreasing those of interferon β, which acts in the opposite direction, favoring the chronicity of the infection) and increasing the immune response of the host due to its ability to amplify the expression of some genes responsible for controlling the immune response and stimulating the lymphocytic activity of the T line [37,38,39]. In fact, some studies on cell lines of ovarian tumors have highlighted the ability of the compound to inhibit cellular proliferation and amplify the efficacy of the cisplatin chemotherapy often used in this pathology [39]. These mechanisms of action have led to the consideration of AHCC for use in the recent COVID-19 pandemic [40].

**Table 2:** Relevant research findings.

Author	Ref.	Description
Laniewski et.al.	[4]	Introduces the concept of a different microbiota in relation to the various anatomical sites of the reproductive system and how it impacts the incidence and outcome of related oncological pathologies
Ravel	[6]	Introduction of the concept of vaginal CST
Bertuccioli	[15]	Collection of the essential characteristics of Lactobacillus crispatus M247
Nene’	[16]	First and most authoritative publication on the correlation between unfavorable CST genetic mutation and ovarian cancer risk
Benerjee	[22]	Thorough assessment and explanation of the ovarian microbiota
Zhang	[30]	Prognostic importance of the presence of intratumoral T-cells and indirect confirmation of the importance of the anti-tumor immune response
Di Pierro	[33]	Lactobacillus crispatus M247 oral administration in papillomavirus-infected women; results of a preliminary, uncontrolled, open trial
Smith	[38]	Confirmation of the action of AHCC in the eradication of persistent HPV thanks to the action of immune stimulation.

## 8. Conclusions

The vaginal and ovarian microbiota appear to be both fully involved in HPV. Therefore, certain factors should be considered in the onset and progression of ovarian tumors, especially for the following conditions:

- The presence of an unfavorable CST;
- The presence/persistence of HPV (especially of high-risk strains);
- Interference with the activity of some tumor suppressor genes (p53);
- Influence on the immune system.

From this perspective, a single or combined treatment with *Lactobacillus crispatus* and/or AHCC could constitute an interesting add-on therapy and preventive tool in subjects with a high-risk profile, assuming that this treatment theoretically overlaps with that used for tumor-related dysbiosis of the uterine cervix, based on the described comparisons. The possible clinical advantages of an intervention, such as the one hypothesized in this paper, could be greater, potentially involving a larger population than an oncological intervention, allowing “general” and “oncological” clinical advantages to be differentiated. The former could include all the conditions of patients with an unfavorable CST at the cervical level (in particular the III and IV) and/or those patients that react positively to HPV dosage or have persistent HPV for some time. The second scenario could consider the conditions of affected patients (with current or previous disease) with an advantage in terms of increasing the efficacy of oncological treatments and/or decreasing their side effects. However, above all, patients considered to be at high risk of ovarian oncological pathologies, such as those with an important family history but who are not candidates for genetic testing (particularly if they are very young) or those with positive but non-surgical tests (refusal of prophylactic surgery or not practicabile due to age) could be included “only” in programs of clinical–instrumental surveillance (Table 3).

The reduction in risk factors, including the modulation of microbial aspects related to the development of the disease, could prove decisive for these patients since, as illustrated, increasing evidence appears to confirm the influence of these scenarios on the concrete clinical expression of mutations. Further clinical studies relating to the effectiveness of individual treatments and their association are necessary to clarify real clinical impacts and plan any future clinical applications in prevention, treatment, and add-on therapy.

## Figures and Tables

**Table 1:** List of all abbreviations.

Abbreviation	Description
AHCC	Active xerose correlated compound
BRCA1	Breast cancer tumor suppressor genes 1
BRCA1	Breast cancer tumor suppressor genes 2
CMV	Cytomegalovirus
CST	Community state type
FRT	Female reproductive tract
HHV-6a	Human herpesvirus -6a
HPV	Human papillomavirus
IGFBPs	Insulin-like growth-factor-binding proteins
SH3RF2	SH3 domain-containing ring finger 2

**Table 3:** General and oncological potential application.

General	Oncological
Patients with unfavorable CST	Affected patients (current or previous)
Patients HPV+ and/or with persistent HPV+	High-risk women

## Data Availability

Not applicable.
